# First report on natural reproduction of rainbow trout *Oncorhynchus mykiss* (Actinopterygii: Salmonidae) in the Okna River (Tisza River basin, Slovakia)

**DOI:** 10.1007/s11756-026-02215-3

**Published:** 2026-06-29

**Authors:** Jakub Fedorčák, Ján Koščo, Kurt Pinter, Libor Závorka

**Affiliations:** 1 Faculty of Humanities and Natural Sciences, Department of Ecology, University of Prešov, Prešov, Slovakia; 2 Institute of Hydrobiology and Aquatic Ecosystem Management, BOKU University, Vienna, Austria; 3 WasserCluster – Biologische Station Lunz, Inter–University Center for Aquatic Ecosystem Research, Lunz Am See, Austria

**Keywords:** Rainbow trout, Aquaculture, Established, Danube basin, Europe

## Abstract

*Oncorhynchus mykiss* is among the most extensively farmed fish species globally and simultaneously ranks as one of the most invasive aquatic taxa. Despite its non-native status in Europe, it remains a valued species in recreational fisheries and is frequently used to supplement streams with declining native *Salmo trutta* populations. In Slovakia, the presence of *O. mykiss* dates back to the late nineteenth century, with historical introductions into the Okna River basin and Lake Vihorlat occurring in the early twentieth century. Here, we present fish stock data and report the first documented occurrence of natural spawning redds of *O. mykiss* in the Okna River recorded in April 2025. This evidence confirms the existence of a self-reproducing, spring-spawning population, possibly descended from the original introductions. The spawning redds were located in gravel-sand substrates in shallow, low-flow areas after the period of rising water temperature and during stable discharge. Stock-assessment data indicate that *O. mykiss* is currently dominant over *S. trutta*, and comparisons with historical records suggest altered relative abundances of *B. barbatula* and *Phoxinus* sp. populations. These findings highlight, ongoing ecological interactions between non-native *O. mykiss* and native fish fauna in the Okna River.

## Introduction

*Oncorhynchus mykiss* Walbaum, 1792 is native to the watersheds of North America and the Kamchatka Peninsula in Russia (Crawford and Muir 2008). During the late 19th and early twentieth centuries, the species was progressively introduced from North America to other regions for aquaculture and recreational purposes. It was first imported into Europe between the late 1870s and early 1880s, particularly in France and Germany (Stanković et al. 2015). Currently, *O. mykiss* is widely distributed across European river basins. Although MacCrimmon (1971) reported that naturalized populations were limited to a few countries, more recent studies suggest an increasing number of established populations throughout the continent (Stanković et al. 2015; Kazakov et al. 2023; Mueller et al. 2025; Pinter et al. 2025).

**Fig. 1:**
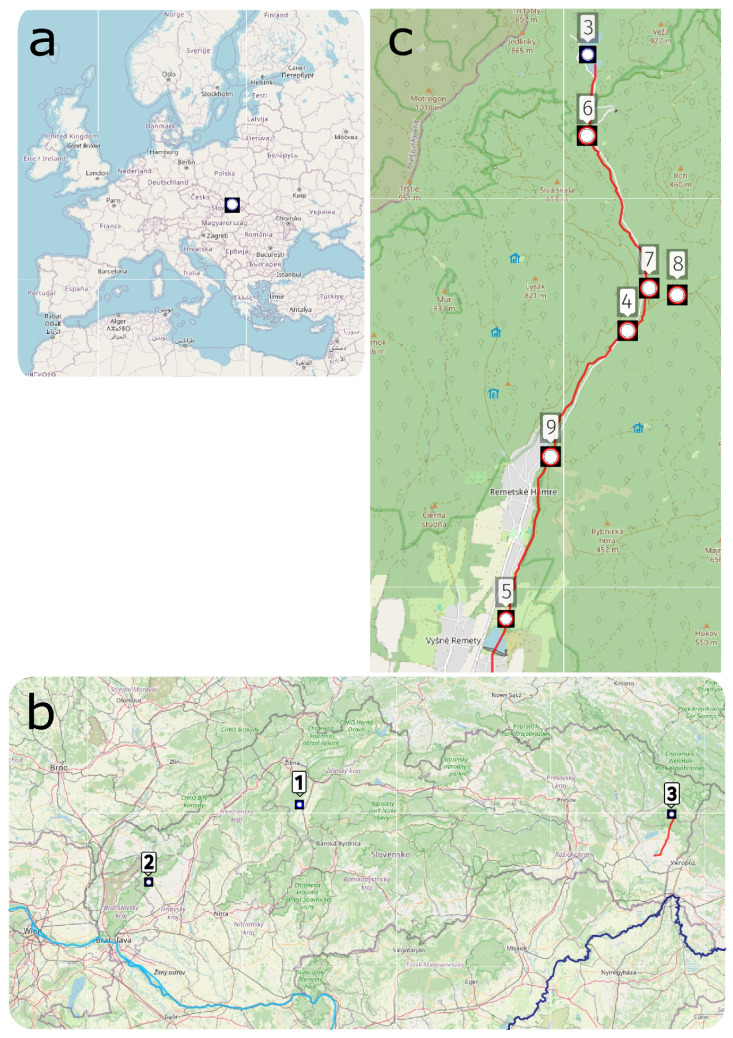
Maps showing the distribution of *O. mykiss*: **a** location in Europe; **b** distribution of the species within Slovakia (light blue line – the Danube River; dark blue line – the Tisa River; red line – the Okna River). Numbers indicate the sites of the first introductions of *O. mykiss*: 1 – tributary of the Váh River near Kláštor pod Znievom; 2 – fish-breeding facility near Smolenice; 3 – Lake Vihorlat. **c** Section of the Okna River with occurrences of the species: site 3 – Lake Vihorlat; sites 4 and 5 are spawning redd habitats; sites 6–9 represent current and historical occurrences of the species (a detailed description of the sites is provided in Table 1)

The earliest introductions of *O. mykiss* to the territory of present–day Slovakia are associated with the activities of aristocratic families during the period of the Austro–Hungarian Empire, in the early 1880s. Baron Révay reportedly imported fertilized *O. mykiss* eggs into a tributary of the Váh River near Kláštor pod Znievom (Fig. 1b, site 1) (Příhoda 2006). Similarly, Count Pálffy established the species in a fish–breeding facility near Smolenice (Fig. 1b, site 2) (MacCrimmon 1971), and Count Károlyi introduced rainbow trout to the Szécsényi estate beneath the Vihorlat Mountains, specifically into Lake Vihorlat (Fig. 1b, site 3) during 1910–1911 (Koščo et al. 2000).

According to Holčík (1989), the introduction of Lake Vihorlat likely involved *O. mykiss* strain from Canada, due to familial ties between the Szécsényi and Vanderbilt families, the latter having origins in Canada. Although the introduction of *O. mykiss* to Lake Vihorlat did not initially meet the owners' expectations (the fish grew to smaller sizes than the native *S. trutta* L., 1758), it led to the establishment of a stable population that spawned in the lake’s tributaries (Koščo et al. 1998). The presence of *O. mykiss* in the lake has been confirmed several times (Holčík 1989; Koščo and Košuth 1998a). Although the lake is of natural origin in the Carpathians, a dam was constructed here in the 1870s to augment the water supply for timber rafting and to operate the water-powered hammer forge in the village of Remetské Hámre (Terek 2009). The dam subsequently experienced water leakage and was reconstructed several times (1928, 1972, 1973, 1981, 1986). As early as the 1960s, *O. mykiss* had already been observed in the Okna River (Holčík and Mišík 1962; Kirka et al. 1980, 1981). Due to suboptimal spawning conditions in the lake’s tributaries and subsequent eutrophication, the rainbow trout stock in Lake Vihorlat gradually declined, while the stock in the Okna River subsequently became more abundant (Koščo and Košuth 1998b).

The primary objective of this study was to assess the species’ distribution, abundance, characterize its length structure, and identify potential spawning redds. In addition, the study compiles and critically evaluates historical introductions and records of the species within the basin, most of which were previously accessible only through grey literature. Although the genetic identity of the Okna River *O. mykiss* stock remains unresolved, preliminary genetic analyses suggest that it may not be genetically uniform and appears distinct from fish farm stock.

## Methods

Basic stream characteristics, including elevation, GPS coordinates, and riverbed slope, were obtained using Google Earth (2025). Substrate composition was visually estimated following the method described by Fedorčák et al. (2018) and partially according to international scale (ISO 14688–1). The water discharge and temperature data were provided by the Slovak Hydrometeorological Institute (SHMI).

Monitoring of the *O. mykiss* stock was carried out in August and October 2024 and in March 2025 under valid fish sampling permit. In total, we surveyed six localities (Fig. 1c, sites 4–9) using certified battery powered electrofishing unit (Hans Grassl IG 200), with segment lengths ranging from 50 to 100 m (Table 1). Standard length of caught fish (SL) was measured in millimeters. A frequency distribution of specimen SL was generated using bin widths of 50 mm. Graphical outputs were created with the using of OpenStreetMap (2025), Inkscape Project (2025), R Statistical Software (R Core Team 2025) and ggplot2 package (Wickham 2016).

**Table 1:** Selected historical and contemporary data on the relative dominance (%) and numbers of *O. mykiss* in the ichthyofaunal community of the Okna River, accompanied by descriptions of the sampling sites. Notes: * this study

Locality	GPS (N,E)	Figure 1c	Authors	Month_year	Segment (m)	*O. mykiss*	*S. trutta*	*P. phoxinus*	*B. barbatula*	*E. danfordi*
Okna R. near Barlahov stream	48.8757236, 22.2102156	4	*	8_2024	100	81.8	18.2			
				10_2024	100	80.5	19.5			
				3_2025	100	87.5	12.5			
				4_2025		spawning redd				
Okna R. near Vyšná Rybnica	48.8310656, 22.1793272	5	*	8_2024	100	23.5	0.9	55.2	19	1.4
				10_2024	100	29.9	4.4	54.7	11	
				3_2025	100	12.6	3.6	62.2	20.7	0.9
				4_2025		spawning redd				
Okna R. below Lake Vihorlat	48.9031122, 22.1976789	6	Holčík and Mišík (1962)	10_1962			75	25		
			Koščo et al. (1998)	8_1998			100			
			*	3_2025	50	50	50			
Okna R. near Barlahov	48.8804122, 22.2117658	7	Holčík and Mišík (1962)	10_1962		23.8	33.3	4.8	33.3	4.8
			Fedorčák and Koščo (2025)	9_1994		15.9	84.1			
			*	3_2025	50	66.7	33.3			
Barlahov stream	48.8793044, 22.2183319	8	Holčík and Mišík (1962)	10_1962		38.1	7.0	21.1	33.8	
			*	3_2025	50	100				
Okna R. near Remetske Hámre	48.8550039, 22.1893667	9	Holčík and Mišík (1962)	10_1962		18.6	14.9	47.1	11.2	8.2
			Fedorčák and Koščo (2025)	9_1994		69.8	24.2		3.0	3.0
			Fedorčák and Koščo (2025)	10_1997		37.5	56.3		6.2	
			Fedorčák and Koščo (2025)	4_2003		57.1	42.9			
			*	3_2025	100	83.3	16.7			

Fish eggs were collected on 25th April, 2025, from two spawning redds located at separate sites along the Okna River: Barlahov (Fig. 1c, site 4) and Rybnica (Fig. 1c, site 5). A standard D–shaped kick net, typically used for benthic macroinvertebrate sampling, was employed for egg collection.

## Results

**Fig. 2:**
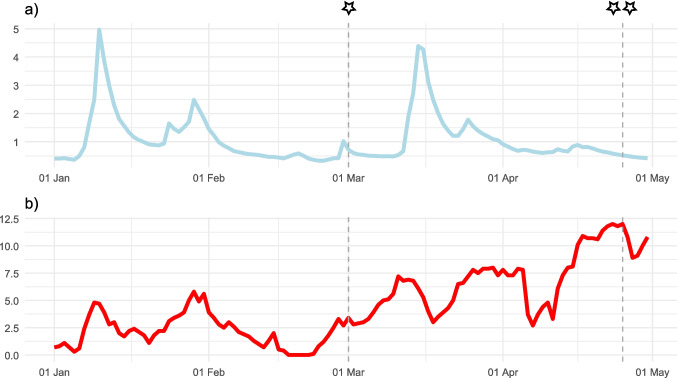
**a** Flow rate in m^3^. s^–1^; and **b** water temperature (°C) recorded between 1st of January and 30th of April 2025 in the Okna River. Marks: one star – March 2025 developed ovaries (Fig. 3a), double star – April 2025 the eggs present in spawning redds (Fig. 3b–d). Data source: Slovak Hydrometeorological Institute (SHMI)

The riverbed slope of the Okna River ranges from 17–21% at the sampling sites Barlahov and Rybnica (Fig. 1c, sites 4, 5). This relatively steep gradient results in the predominance of coarse substrates, primarily boulders and cobbles. Suitable spawning habitats containing finer sediments (gravel, sand) are limited and tend to become available mainly during the spring, when the littoral zone is inundated (pers. obs.). Two weeks preceding egg collection (25th April 2025), the water temperature in the Okna River increased from 3.3 °C to 12 °C (SHMI water gauge station), while discharge ranged from 0.52–0.89 m^3^·s⁻^1^ (Fig. 2).

The ichthyocenosis of the Okna River is composed of *O. mykiss*, *S. trutta*, *Barbatula barbatula* L., 1758, *Phoxinus* Rafinesque, 1820 sp., and *Eudontomyzon danfordi* Regan, 1911 (Table 1).

Spatial variation in species composition was evident: at the Barlahov locality, only two species were detected (Table 1, site 4), whereas all five species occurred at the Vyšná Rybnica locality (Table 1, site 5). The relative abundance of *O. mykiss* across the surveyed sites ranged from 12.6–87.5% (Table 1). Length-frequency distributions of *O. mykiss* individuals from the Okna River are provided in Table 2.

**Table 2:** Length-frequency (SL, mm) distribution of *O. mykiss* at two sampling locations in the Okna River

Locality	Month_Year	0–49	50–99	100–149	150–199	200–249	Mean SL (mm)
Barlahov	August_24	5	12	17	1	1	102.5
	October_24	7	16	8	2	0	85.2
	March_25	2	5	0	0	0	53.4
Rybnica	August_24	1	12	12	6	1	112.9
	October_24	2	12	10	5	1	108
	March_25	0	5	4	4	1	131.6
Total		17	62	51	18	4	102.5

**Fig. 3:**
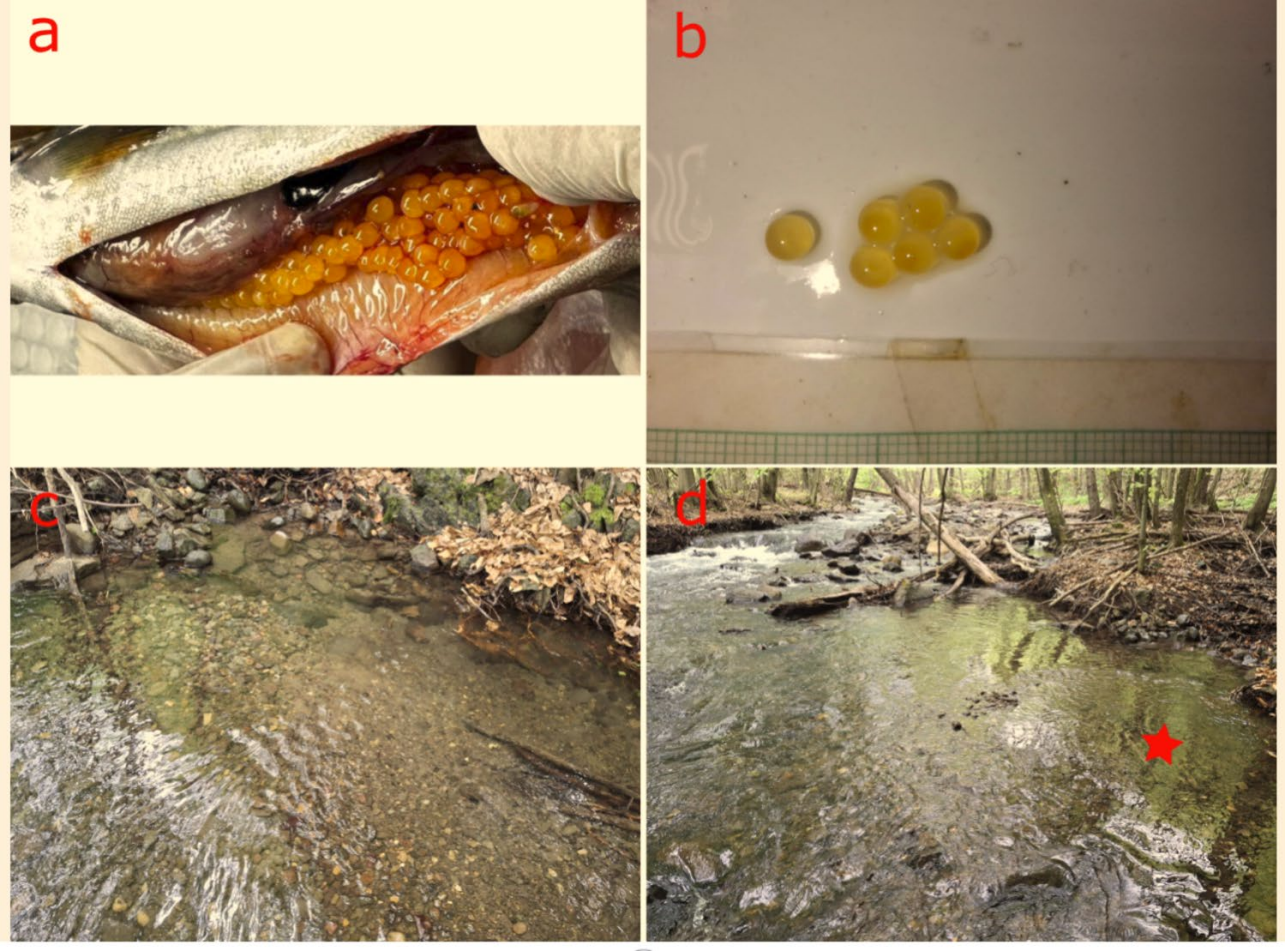
**a** Developed ovaries of *O. mykiss* female collected in March 2025 (locality Rybnica); **b** eggs of *O. mykiss* collected in April 2025; **c** gravelly and sandy substrate of the spawning redd; **d** locality Barlahov (Fig. 1c, site 4)—location of the spawning redd indicated by red star

Female *O. mykiss* observed during the March 2025 survey exhibited mature gonads (Fig. 3a).

**Fig. 4:**
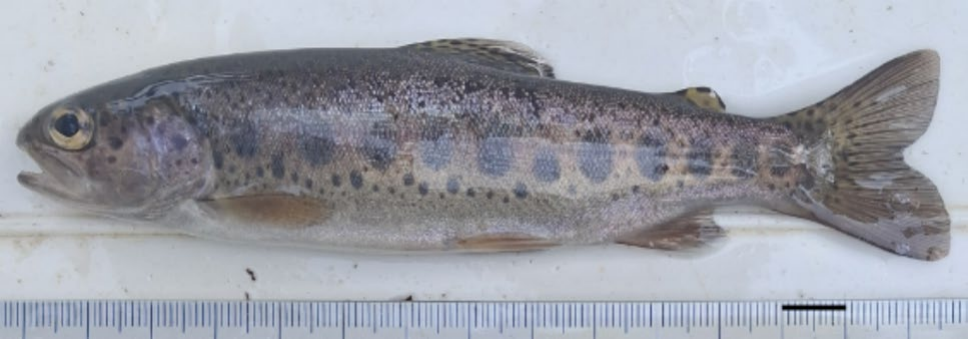
The morphotype of *O. mykiss* from the Okna River (black line represent 1 cm)

The spawning redds with eggs were subsequently confirmed on April 25, 2025 during benthic sampling at two sites (Barlahov, Rybnica) along the Okna River. The fertilization of eggs and developmental stages from spawning redds has not been investigated. Eggs (Fig. 3b) were collected from two spawning redds located in areas with gravelly–sandy substrate (Fig. 3c). The redds were situated outside the main current, in locations characterized by diagonally oriented water flow (Fig. 3d). Each sampling area measured approximately 0.5 × 0.5 m, with an average water depth of 20 cm. Based on the recorded species, we can conclude that the eggs found in spawning redds during April of 2025 belong to *O. mykiss*. Overall, the spawning redds were small and poorly visible, likely due to the small body size of spawners (Table 2, mean SL = 102.5 mm). A representative specimen of *O. mykiss* from the Okna River is shown in Fig. 4.

## Discussion

The Okna River basin is located in the Vihorlat Mountains, which are of volcanic origin – stratovolcano (Olekšák et al. 2014). The *O. mykiss* population in the Okna River basin most likely originates from Lake Vihorlat – the source of the Okna River. The initial introductions (1910–1911) probably involved specimens imported from Canada (Holčík 1989; Koščo et al. 2000). Species subsequently spawned in the lake’s tributaries during spring (Koščo et al. 1998), additionally, the authors observed juveniles near the lake’s tributaries during autumn sampling. The most recent published record of *O. mykiss* in Lake Vihorlat dates to the 2003 (Fedorčák and Koščo 2025).

The earliest published records of *O. mykiss* in the Okna River date back to the 1960s (Holčík and Mišík 1962) and reported abundance ranged from 18–38% (Table 1). Subsequently, the occurrence of the species was confirmed by Koščo and Košuth (1998b) in the Okna River section near the village of Remetské Hámre with a dominance of 37.5%. Fedorčák and Koščo (2025) documented *O. mykiss* historical occurrences in the Okna River near Remetské Hámre, Vyšná and Nižná Rybnica villages during 1994, 1997, 1998, 2000, 2003, 2015, and 2021. *Oncorhynchus mykiss* occurred throughout the Okna River, from its source (Fig. 1c, site 3) to the Vyšná Rybnica reservoir (Fig. 1c, site 5), representing 12.6–100% of the fish community (Table 1). In comparison with data from the 1960s (Holčík and Mišík 1962), our findings suggest that the relatively high abundance of *O. mykiss* is likely influencing the abundances of *S. trutta*, *B. barbatula*, and *P. phoxinus* within the surveyed sections (Table 1). The abundance dynamics of *O. mykiss* in the Okna River may also be influenced by seasonal factors, such as spawning migrations, which should be verified in future studies. In summary, there are indications that the species may have been present in the Okna River for over 60 years at the relatively high population density (Table 1) and in various size classes (Table 2). Along with the finding of spawning redds and eggs (Fig. 3b–d) the results of this work indicate a self–sustaining population isolated in relatively short section of the Okna River (c. 10 km) and in its smaller tributaries between Lake Vihorlat and Vyšná Rybnica reservoir.

Currently, a private trout farm and hatchery operate within the Okna River basin. The breeding facilities are not directly connected to the Okna riverbed, and there are no channels that would allow hatchery *O. mykiss* to escape into the stream. Additionally, the Okna River functions as a managed fishing ground administered by the Forests of the Slovak Republic (FSR) and the Slovak Angling Association (SAA). Stocking of *O. mykiss* in Slovak fishing grounds is typically seasonal (spring) and linked to the “trout fishing season”. During this period, stocking is authorized by the Ministry of the Environment of the Slovak Republic for the purposes of recreational fishing by SAA members. Standardly, the stocked individuals of *O. mykiss* for angling purposes meet length limits regulation (TL = 270 mm, Decree No. 381/2018), but the specimens of this lengths do not typically occur in the Okna River (Table 2). At present, no official records of *O. mykiss* stocking currently exist for the Okna River, *S. trutta* is regularly stocked (information from SAA).

An overview of potential and confirmed established populations of *O. mykiss* in Europe was provided by MacCrimmon (1971) and Stanković et al. (2015). Since then, additional established populations have been reported in Crete (Stoumboudi et al. 2017), Bulgaria (Kazakov et al. 2023), Germany (Mueller et al. 2025), Austria (Pinter et al. 2025), and Cyprus (Spairani et al. 2025). The authors state that most of established populations reproduce during the spring at water temperatures between 5.2 and 12 ºC which is in line with our observation from the Okna River (Fig. 2b). It is generally accepted, that size and depth of spawning redd is influenced by the body length and body height of the trout female which constructing the redd (Raleigh et al. 1984). In line with this relationship, spawning redds of Okna rainbow trout were poorly visible and occurred in relatively shallow river sections (c. 20 cm depth; Fig. 3c, d), which may correspond to the mean standard length (SL) of the recorded specimens (Table 2). Similarly, relatively small spawning redd dimensions (mean length 40.5 cm; mean width 34.2 cm) were reported by Candiotto et al. (2011) in Italian stream, where spawning females had an average body length of 261.4 mm.

Although the historical background of *O. mykiss* in the Okna River basin is well documented, the genetic identity of this population remains unresolved. Therefore, we recommend conducting molecular analyses to clarify the genetic status of *O. mykiss* stock in the Okna River. Additionally, enhanced monitoring efforts should focus on detecting potentially established *O. mykiss* populations in other Slovak river basins. Considering the co-occurrence of native species such as the *S. trutta* in the Okna River, further research is warranted to investigate possible competitive or predatory interactions among these taxa.

## Data Availability

The data can be provided by the corresponding author on a reasonable request.
